# Broadband Solar Metamaterial Absorbers Empowered by Transformer‐Based Deep Learning

**DOI:** 10.1002/advs.202206718

**Published:** 2023-02-28

**Authors:** Wei Chen, Yuan Gao, Yuyang Li, Yiming Yan, Jun‐Yu Ou, Wenzhuang Ma, Jinfeng Zhu

**Affiliations:** ^1^ Institute of Electromagnetics and Acoustics and Key Laboratory of Electromagnetic Wave Science and Detection Technology Xiamen University Xiamen Fujian 361005 P. R. China; ^2^ Shenzhen Research Institute of Xiamen University Shenzhen Guangdong 518057 China; ^3^ Optoelectronics Research Centre and Centre for Photonic Metamaterials University of Southampton Highfield Southampton UK SO17 1BJ; ^4^ State Key Laboratory of Electronic Thin Films and Integrated Devices National Engineering Research Center of Electromagnetic Radiation Control Materials Key Laboratory of Multi‐spectral Absorbing Materials and Structures of Ministry of Education University of Electronic Science and Technology of China Chengdu Sichuan 610054 P. R. China

**Keywords:** absorbers, deep learning, metamaterials, solar energy, transformers

## Abstract

The research of metamaterial shows great potential in the field of solar energy harvesting. In the past decade, the design of broadband solar metamaterial absorber (SMA) has attracted a surge of interest. The conventional design typically requires brute‐force optimizations with a huge sampling space of structure parameters. Very recently, deep learning (DL) has provided a promising way in metamaterial design, but its application on SMA development is barely reported due to the complicated features of broadband spectrum. Here, this work develops the DL model based on metamaterial spectrum transformer (MST) for the powerful design of high‐performance SMAs. The MST divides the optical spectrum of metamaterial into *N* patches, which overcomes the severe problem of overfitting in traditional DL and boosts the learning capability significantly. A flexible design tool based on free customer definition is developed to facilitate the real‐time on‐demand design of metamaterials with various optical functions. The scheme is applied to the design and fabrication of SMAs with graded‐refractive‐index nanostructures. They demonstrate the high average absorptance of 94% in a broad solar spectrum and exhibit exceptional advantages over many state‐of‐the‐art counterparts. The outdoor testing implies the high‐efficiency energy collection of about 1061 kW h m^−2^ from solar radiation annually. This work paves a way for the rapid smart design of SMA, and will also provide a real‐time developing tool for many other metamaterials and metadevices.

## Graphical abstract

This work develops the deep learning model based on metamaterial spectrum transformer (MST) for the powerful design of high‐performance solar metamaterial absorbers. The MST divides the optical spectrum of metamaterial into *N* patches, which overcomes the severe problem of overfitting in traditional deep learning model and boosts the learning capability significantly.

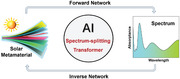

## 1 Introduction

In the past few years, the investigations of metamaterial absorbers have attracted a surge of interest due to their extensive applications in radiative cooling, photovoltaics and solar‐thermal energy harvesting.^[^ ^1^, ^2^, ^3^, ^4^ ^]^ Among these studies, solar metamaterial absorbers (SMAs) demonstrate great potential for converting sunlight into available green energy, which can be widely applied in thermophotovoltaics, seawater desalination, and sewage purification.^[^ ^5^, ^6^, ^7^ ^]^ In the past decade, various SMAs have been investigated for high broadband absorption of sunlight. In general, they consist of multiple subwavelength thin film layers due to the simple high‐throughput nanofabrication.^[^ ^8^, ^9^ ^]^ Lin et al. have demonstrated an SMA with graphene/dielectric multilayers.^[^ ^10^ ^]^ Wu et al. have proposed an SMA with alternative tungsten/silica multilayer thin films.^[^ ^11^ ^]^ Such alternating nanostructures usually require the optically‐thin‐enough (1–10 nm) layers of lossy materials to trap light sufficiently, which inevitably increases fabrication complexity and partially hinders thermal conduction. In order to overcome this problem, one can consider another light absorbing scheme, i.e., the design of graded‐refractive‐index (GRI) thin dielectric layers on the metallic mirror.^[^ ^12^, ^13^ ^]^ However, it is a great challenge to design such SMAs within a short time. Conventionally, it relies on broadband impedance matching in the entire solar spectrum, and this demands numerous experiments, optical simulations, and some classical algorithms to optimize layer thicknesses.^[^ ^14^, ^15^ ^]^ These algorithms are always brute‐force and even confined to the local optimal solutions.^[^ ^16^ ^]^ Therefore, it is still quite essential to pursue a powerful method for designing broadband SMAs by the GRI multilayer scheme.

In the past few years, the design field of nanophotonics and metamaterials has received a great developing opportunity by using deep learning (DL),^[^ ^17^ ^]^ because DL can significantly shorten the time‐consuming simulation process and provide flexible design paradigms for various applications. Many global leading scientists in the field of nanophotonics and metamaterials have expressed their optimism and perspective on DL for boosting the related scientific studies,^[^ ^18^, ^19^, ^20^ ^]^ which inspire a lot of researchers with great interest. Up to date, researchers have exploited a series of full‐blown neural networks (NNs) to energize the metamaterial design, such as multilayer perceptron (MLP), convolutional neural network, recurrent neural network, generative adversarial networks, variational autoencoder, transfer learning, and few‐shot learning.^[^ ^21^, ^22^, ^23^, ^24^, ^25^, ^26^, ^27^, ^28^, ^29^ ^]^ Very recently, a state‐of‐the‐art artificial network named transformer is extraordinarily blooming in the field of DL and has been widely used in natural language processing and computer vision.^[^ ^30^, ^31^ ^]^ It has shown the dominant learning capability by the unique positional encoding and multihead attention mechanism, which draws global dependencies between input and output and allows for significantly more parallelization. Despite this, the use of a conventional transformer (CT) on a broadband optical spectrum of metamaterial with a significant number of wavelength points will lead to a dramatical increase of network parameters and could make it difficult for a network to converge and generalize well.^[^ ^32^ ^]^ Therefore, a metamaterial spectrum transformer (MST) architecture for the smart design of SMAs is still in demand.

Here, we develop an MST by segmenting the input optical spectrum of metamateiral and accomplish the high‐efficiency design of SMAs based on a GRI scheme. Compared with the CT in the inverse neural network (INN), the MST demonstrates much better performance on model training and can learn various physical concepts. It also uses less training parameters and shows a much better prediction accuracy than the classical MLP model in the forward neural network (FNN). A universal tool with the customer freely defined dots is developed to facilitate the progress of SMA inverse design. Based on the previous investigation, we fabricate a wafer‐scale SMA with broadband sunlight absorptance designed by MST. The on‐site experiments under the sunlight indicate that our SMA is promising for photothermal conversion. Our study provides a way to develop SMAs and various other metamaterial‐based applications via transformer‐based artificial intelligence.

## 2 Results and Discussion

### 2.1 Smart Design of GRI‐Based SMAs by Data‐Driven MST

**Figure 1:**
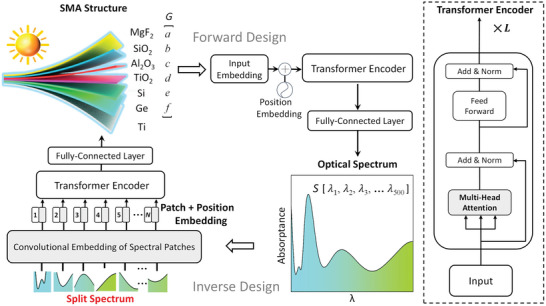
Schematic drawing of metamaterial spectrum transformer (MST) architecture for the smart design of graded‐refractive‐index (GRI) based solar metamaterial absorbers (SMAs).

Our SMA design adopts the subwavelength GRI multilayer with a Ti substrate for broadband sunlight absorption, as shown in **Figure** **1**. In fact, subwavelength multilayer structures are usually used as the classical paradigm to evaluate state‐of‐the‐art DL models for the design of metamaterials and nanophotonics.^[^ ^17^, ^33^, ^34^ ^]^ The GRI layers consist of MgF_2_, SiO_2_, Al_2_O_3_, TiO_2_, Si, and Ge from top to bottom. In the solar spectral range, their real parts of the complex refractive index increase gradually, which is generally in accordance with the impedance gradation principle from the air to the metamaterial. In order to accomplish the intelligent design of GRI‐based SMAs, we build up the MST network consisting of the inverse design and forward design, as illustrated in Figure 1. The inverse design is used to predict the SMA structure parameters according to the input of spectral response. The forward design is adopted to take the place of time‐consuming simulation and confirm the effectiveness of inverse design immediately.

In the inverse design, the input corresponds to the spectral vector ***S*** for 500 wavelength points from 300 to 2500 nm, while the output is the GRI‐based metamaterial vector ***G***, where *a, b, c, d, e*, and *f* represent the thicknesses of GRI layers. With the aim to utilize the advantages of the transformer encoder, we overcome the significant dimension mismatch between the input ***G*** and output ***S*** by using an input‐splitting scheme. In this scheme, we divide the spectrum into 25 patches, perform one‐dimensional convolutional embedding on each of them, which can transform the spectrum patches into a vector representation that can be processed by the MST model. In order to make use of the order of spectrum sequence, we add position embeddings to inject the positional information of spectrum. We feed the resulting sequence of vectors to a standard transformer encoder. Such an encoder consists of *L* identical layers. Each layer has two sublayers, including multihead attention (with *M* degrees of freedom) and a position‐wise feed‐forward network. Each sublayer is surrounded by a residual connection, followed by layer normalization. In view of the multihead attention, we adopt the following equation to describe the self‐attention mechanism,^[^ ^30^ ^]^

(1) $$\mathrm{Attention}\;\left(Q,K,V\right)=\;\mathrm{softmax}\left(\frac{QK^{T}}{\sqrt{d_{k}}}\right)V$$

where *Q*, *K*, and *V* are the query matrix, key matrix, and value matrix, respectively. They are all obtained from the linear transformation of the input sequence and *d*_k_ is the length of these matrices. We calculate the dot products of *Q* with all the keys, dividing each by$\;\sqrt{d_{k}},$ and use the softmax function to get the weights on the values. The multihead attention mechanism obtains different representations of (*Q*, *K*, and *V*), which can produce different attention patterns for the heads on each layer. Each head attention can be described as below,^[^ ^30^ ^]^

(2) $${\;\mathrm{head}}_{i}=\;\mathrm{Attention}\left(QW_{i}^{Q},KW_{i}^{K},VW_{i}^{V}\right)$$

(3) $$\begin{array}{ccc} & & \mathrm{MultiHead}\;\left(Q,K,V\right)\\ & & =\;\mathrm{Concat}\;\left({\mathrm{head}}_{1},\text{…}{\mathrm{head}}_{i},\text{…}{\;\mathrm{head}\;}_{M}\right)W^{O} \end{array}$$

where the $W_{i}^{Q}$, $W_{i}^{K}$, and $W_{i}^{V}$ are weight parameter matrices for *Q*, *K*, *V*, respectively. There are *M* heads for the attention mechanism. The ${\mathrm{head}}_{i}$ represents the *i* ^th^ head attention. *W* ^O^ denotes the weight parameter matrix related to all the heads. By calculating each head attention and concatenating the results as Equation (3), we can get the multihead attention mechanism results. The second sublayer is a fully connected feed‐forward network, which is applied to each position separately and identically. By adding a fully connected layer after the transformer encoder, we can obtain the predicted SMA structure parameters. In the forward design FNN, the input is structure parameters vector ***G***, we will also perform input embedding on each structure parameter, add position embedding, and feed the resulting sequence of vectors to a standard transformer encoder which is composed of a stack of *L* identical layers. We can get the predicted optical response *S* after a fully connected layer. In general, the entire DL model adopts the ReLU^[^ ^35^ ^]^ as an activation function. The Adam is used as the gradient descent optimizer.^[^ ^36^ ^]^ The loss function is based on the calculation of mean square error (MSE) between the predicted results and the ground truth from optical simulation, which is defined as the following equation,^[^ ^37^ ^]^

(4) $$\mathrm{Loss}\;=\frac{1}{N}\;\sum_{i\;=\;1}^{N}{\left({S^{\prime }}_{i}-{\tilde{S}}_{i}\right)}^{2}$$

where $S_{i}^{\prime }$ is the spectrum or metastructure predicted by the DL models, ${\tilde{S}}_{i}\;$is the simulated spectrum or metastructure, and *N* is the number of samples.

### 2.2 Model Performance of MST

**Figure 2:**
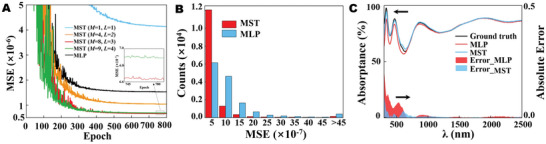
A) Learning curves of multilayer perceptron (MLP) and metamaterial spectrum transformer (MST) with different *M* and *L* values. B) Mean square error (MSE) distributions of testing samples. C) Predicted spectra and the comparison of their prediction errors.

We first evaluate the MST performance in FNN and compare it with the classical MLP model (see more details in Figure S1, Supporting Information). As illustrated in **Figure** **2**A, the MST model can only extract a few features when *M* and *L* are 1. With the increase of parameters *M* and *L*, it is observed that the MSE gets lower. The use of *M* = 8 and *L* = 3 generates the minimum MSE as shown in Figure 2A. The MSE would become larger when *M* and *L* continue to increase due to the overfitting and gradient vanishing^[^ ^38^, ^39^, ^40^ ^]^ (More details are discussed in Supporting Information). The MST with the optimized network configuration (*M* = 8 and *L* = 3) demonstrates about half of the MSE (≈6.63 × 10^−7^) for MLP by using less training parameters (see more details in Figure S2 and Table S1, Supporting Information). The histogram of MSE count distributions in Figure 2B illuminates that the MST supports more testing instances with a lower MSE than the MLP model. Specifically, the MST model has 84.8% samples for MSE ≤ 5×10^−7^ and much less samples for MSE > 4.5×10^−6^. This property implies the higher prediction accuracy of MST in the entire test sampling space. In Figure 2C, we randomly pick up an MST‐predicted spectrum and provide a comparison with the ground truth and MLP prediction. The MST model evidently reduces the absolute spectral errors over the entire wavelength range. Particularly, the absolute errors are dramatically decreased surrounding the prominent spectral dips and peaks in the range of short wavelengths. This advantage is very beneficial for the precise design of SMA since the sharp spectral features are common in metamaterial design and the majority of the solar energy is distributed in this short‐wavelength range. Particularly, our method not only can get electromagnetic responses immediately for multilayer film but also provides a way to calculate electromagnetic responses of various other metamaterial‐based applications (see more details in Supporting Information).

**Figure 3:**
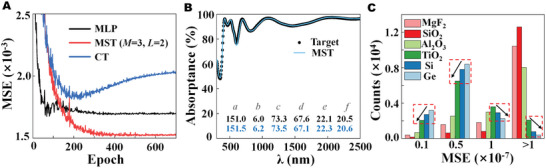
A) Comparison of learning curves for multilayer perceptron (MLP), metamaterial spectrum transformer (MST), and conventional transformer (CT) models. B) A randomly selected sample with a precise prediction from testing samples. C) Mean square error (MSE) count distributions of all six geometry parameters in the testing samples.

With the aim to investigate the performance of MST in INN, we compare its learning curve with the MLP and CT models in **Figure** **3**A. Obviously, the learning curve of CT has a problem of overfitting, while the MLP and MST can converge to a constant minimum MSE. Specifically, the MST converges to the lowest MSE of ≈1.52 × 10^−3^ within the testing instances, although it takes a much smaller number of training parameters than the other two models (see Figure S2 and Table S2, Supporting Information). The MST has a revolutionary learning capability inherited from CT and demonstrates better prediction performance than MLP. This is mainly attributed to our unique spectrum‐splitting scheme in the INN. The spectrum‐splitting scheme overcomes the serious mismatch from the large‐dimension vector *S* (1 × 500 input) to the small‐dimension vector *G* (1 × 6 output) in a CT architecture, which inevitably raises the complexity of the training network and leads to overfitting. In order to exhibit the prediction accuracy of MST in INN, we randomly pick up an instance, as illustrated in Figure 3B. Compared with the ground truth, the absolute prediction errors of all six geometry parameters are less than 3.3%. Such small errors make the two spectra from geometry prediction and ground truth almost the same. We further compare the MSE distribution counts of six material layer thicknesses in Figure 3C. Among these materials, there are two categories: materials whose refractive indexes are less than 1.8 ($n_{{\mathrm{MgF}}_{2}}\;$<$\;n_{{\mathrm{SiO}}_{2}}$< $n_{{\mathrm{Al}}_{2}O_{3}}$) and materials with a refractive index of more than 2.4 ($n_{{\mathrm{TiO}}_{2}}\;$< *n*_Si_ < *n*_Ge_). For the latter, the result indicates a remarkable statistical correlation between the MSE distribution counts and the refractive index. In view of the MSE counts in a smaller range (≤0.5 × 10^−3^), the Ge layer thicknesses take the largest numbers, while the Si layer thicknesses and TiO_2_ layer thicknesses take the second and third place, respectively. It implies that a larger refractive index supports a higher prediction precision. On the contrary, the statistical correlation is not reflected on the materials with a refractive index less than 1.8, and the thickness prediction errors of MgF_2_, SiO_2_, and Al_2_O_3_ are obviously much larger than those of high‐index materials. Notably, the MSE distribution counts of MgF_2_ and SiO_2_ layer thicknesses are about one order larger than those of other material layer thicknesses in the case of MSE ≥ 1 × 10^−3^.

**Figure 4:**
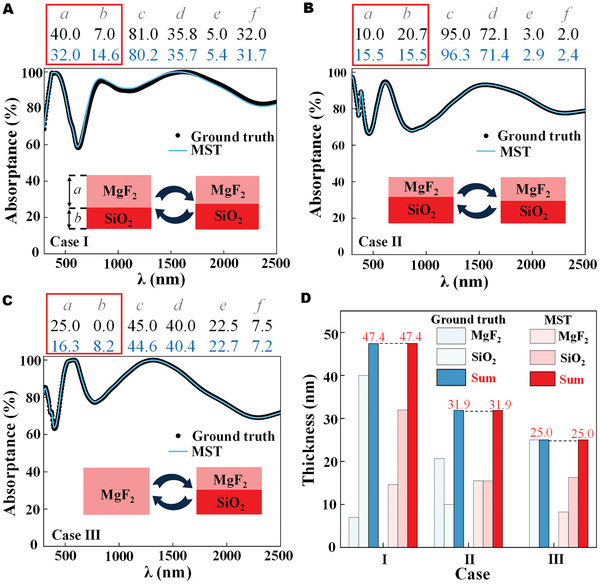
A–C) Inverse design instances randomly picked up from the testing sample space, which illustrates the physical equivalence of our solar metamaterial absorber (SMA) model. D) Thickness physical equivalence of MgF_2_ and SiO_2_ layers learned by the metamaterial spectrum transformer (MST).

We next manage to find out the reason for higher prediction errors on low‐index material thicknesses. Three predicted instances randomly picked up from the testing samples are provided in **Figure** **4**. All of them indicate relatively accurate predictions for the thicknesses of Al_2_O_3_, TiO_2_, Si, and Ge. In contrast, they demonstrate significant predicting deviations on the thicknesses of MgF_2_ and SiO_2_ layers, respectively. Despite this, we observe that the predicted sum of SiO_2_ and MgF_2_ layer thicknesses is almost the same as the ground‐truth sum of MgF_2_ and SiO_2_ layer thicknesses. We further calculate and compare the optical spectra from ground truth and MST. Although there are significant differences in MgF_2_ and SiO_2_ layer thicknesses, the two sets of spectra show striking agreement. The spectral consistency could be attributed to the SMA model's physical equivalence based on the transmission matrix theory of light waves in different material layers.^[^ ^41^ ^]^ Such unique equivalence effects of MgF_2_ and SiO_2_ layer thicknesses are illustrated in the insets from Figure 4A–C. In view of the MgF_2_ and SiO_2_ layers, the equivalent condition for optical propagation can be calculated using the following equation (see Figure S3, Supporting Information),^[^ ^42^ ^]^

(5) $$a+b\times n_{{\mathrm{SiO}}_{2}}/n_{{\mathrm{MgF}}_{2}}=a_{p}+b_{p}\times n_{{\mathrm{SiO}}_{2}}/n_{{\mathrm{MgF}}_{2}}$$

where *a* and *b* are the ground‐truth thicknesses of MgF_2_ and SiO_2_ layers, respectively. *a*_p_ and *b*_p_ are MST‐predicted thicknesses of MgF_2_ and SiO_2_ layers, respectively. Figure 4D illustrates that the MST‐predicted results fit Equation (5) very well, and the MST network has a good capability to learn this physical principle effectively. More importantly, Figure 4C demonstrates that the MgF_2_ layer can totally replace the influence of the SiO_2_ layer on the optical spectrum via the physical equivalence denoted in Equation (5), which also gives a guide to simplify experiments. In order to avoid fabrication complexity, we can adopt only the MgF_2_ layer for the next experiment.

### 2.3 Design of High‐Performance SMA and Physical Analysis

We continue to evaluate our network on the quick and smart design of high‐performance SMA.

**Figure 5:**
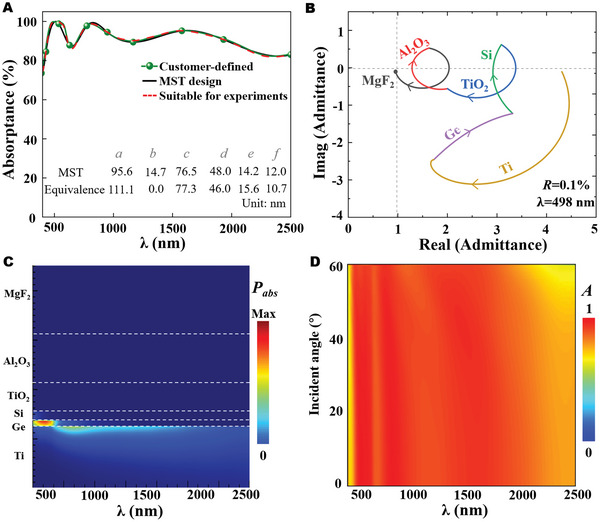
A) Absorptance spectra of the customer‐defined, metamaterial spectrum transformer (MST) predicted and equivalent structures (suitable for experiments), and their geometric parameters. B) Optical admittance locus of the designed solar metamaterial absorber (SMA) at *λ* = 498 nm. The curves with different colors represent the admittance trajectories of each material layer. C) Absorbed power distribution of the designed SMA. D) Absorptance spectrum with different incident angles under an unpolarized source.

In order to generate the on‐demand spectrum more flexibly, we develop the customer freely defined multiple dots in the layout of the spectrum, which supports the cubic interpolation to provide the target spectrum as the input of the inverse design. Since the prediction time is at the millisecond scale after the training of the entire network, the MST can output the real‐time structural parameters according to customer‐defined spectra (see more details and Movie S1, Supporting Information). After a series of systematical investigations, we take a customer‐defined spectrum with exceptional broadband solar absorptance, which requires the computational efforts of about a few days by complicated computational simulations. In contrast, we obtain the corresponding predicted SMA structure parameter values by the MST model within a short time (see more details and Movie S2, Supporting Information). The designed structure and corresponding spectrum are provided in **Figure** **5**A. The MST‐predicting spectrum shows good agreement with the customer‐defined spectrum. According to the above discussion, the predicted MgF_2_ and SiO_2_ layered structure can be simplified by a single layer of MgF_2_ by the equivalence law suggested by MST. Therefore, we can adopt an MgF_2_ layer of 111.1 nm thick instead of the MgF_2_ and SiO_2_ combination layer. We set these thicknesses to the numerical value which would be easily fabricated and the paradigmatic SMA consists of Ti (100 nm)/Ge (10 nm)/Si (15 nm)/TiO_2_ (45 nm)/Al_2_O_3_ (80 nm)/SiO_2_ (0 nm)/MgF_2_ (110 nm). Figure 5A indicates that the SMA structure (suitable for experiments) not only reduces the layer number but also exhibits a spectrum consistent with the target one. The designed structure shows a high average absorptance of over 92% in a broad band from 400 to 2500 nm. Furthermore, we calculate the solar absorption rate *α*_sol_ aiming at illustrating the performance about *I*_AM1.5_ (AM 1.5, ASTM G173‐03, ISO),^[^ ^43^ ^]^

(6) $$\;\alpha_{\mathrm{sol}}=\frac{\int_{400\mathrm{nm}}^{2500\mathrm{nm}}A\left(\lambda \right)\;\times \;I_{\mathrm{AM}1.5}\left(\lambda \right)d\lambda }{\int_{400\mathrm{nm}}^{2500\mathrm{nm}}I_{\mathrm{AM}1.5}\left(\lambda \right)d\lambda }$$

Here, *α*_*sol*_ is as high as ≈0.92, which should be more competitive than many other SMA with complicated metaunits and would facilitate more photothermal applications.^[^ ^44^ ^]^

In order to illuminate the high broadband absorption, we adopt the analysis of optical admittance on the proposed SMA. The normalized complex optical admittances of subwavelength layers are defined by *Y* =$\sqrt{\varepsilon /\mu }$, where *ε* and *µ* denote the relative permittivity and relative permeability of layer material, respectively.^[^ ^45^ ^]^ In the complex coordinate system, the number of (1,0) represents the optical admittance of free space. The high broadband absorption is originated from the superior admittance matching for each working wavelength. Here, we take the wavelength of 498 nm as a representative example, with almost the largest solar radiation intensity. We plot all the layer admittances at the wavelength of 498 nm in Figure 5B. In the beginning, a bare Ti layer demonstrates the complex admittance of (4.31, −0.09), mismatching severely with the free space admittance. The scheme of GRI‐based subwavelength layers introduces a series of additional optical admittances. This introduction leads to the change of equivalent complex admittance at the air/MgF_2_ to (0.97, −0.08), which is very close to the free space admittance of (1,0) in the complex plane. On this condition, we can assume that the complex admittance of SMA is almost perfectly matched with that of the free space, and the reflectance is as low as 0.1% (see more details in Figure S8, Supporting Information). Since the Ti layer is optically thick and opaque, the ultralow reflectance indicates the high absorption performance of the SMA. Furthermore, we investigate the distributions of absorbed power in the entire SMA by full‐wave optical simulation. The absorbed power is calculated by $\;P_{\mathrm{abs}}=\frac{1}{2}\;\omega \varepsilon^{\prime \prime }{\left|E\right|}^{2}$, where *ω* is the angular frequency of incident light, *ε*′′ is the imaginary part of material permittivity, and |*E*| is the electric field intensity.^[^ ^46^ ^]^ Figure 5C illuminates that the *P*_abs_ is almost zero in the layers of MgF_2_, Al_2_O_3_, and TiO_2_ and is very slight in the visible range for the Si layer. Most of the power loss is concentrated in the Ge and Ti layers. Particularly, the visible power loss is predominantly in the Ge layer, whereas the near‐infrared loss is mainly within the Ti layer. This is attributed to the high extinction coefficients of Ge and Ti in the visible and near‐infrared ranges, respectively. We next investigate the absorptance spectrum with different incident angles under an unpolarized source. Figure 5D presents a well angular tolerance feature up to 60 degrees with a slight absorptance variation in the wavelength range of 2000 to 2500 nm. Particularly, the absorptance from 400 to 2000 nm stays almost the same and the average absorptance over the entire range is ≈86% when the incident angle is 60° (see more details in Figure S9, Supporting Information).

### 2.4 Fabrication, Characterization, and Optical Measurement of the SMA

**Figure 6:**
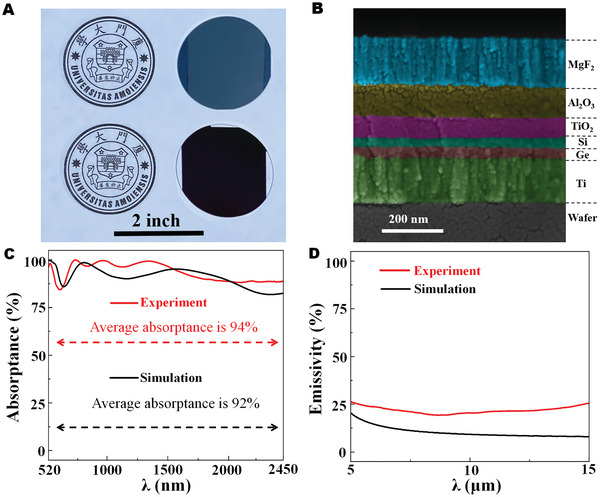
A) Photograph for two types of solar metamaterial absorbers (SMAs). B) Color‐coded scanning electron microscope (SEM) image of the fabricated graded‐refractive‐index (GRI) based SMA. Simulated and measured results for C) absorptance and D) emissivity spectra.

Based on the above design study, we fabricate the SMA sample and perform the characterization and optical measurement. The wafer‐scale SMA samples are shown in **Figure** **6**A. It is a uniform multilayer consisting of MgF_2_, Al_2_O_3_, TiO_2_, Si, Ge, and Ti layers from top to bottom, which looks much darker than the reference of Ti‐coated wafer under the sunlight. We further observe its scanning electron microscope (SEM) cross‐section view in Figure 6B. The thin film boundaries of different materials can be clearly identified, despite the slight interface roughness. It is worth mentioning that the interface roughness is typically influenced by the fabrication process, which is usually not considered in the optical simulation. The measured and simulated optical absorbance spectrum of the SMA sample are plotted in Figure 6C. It can be seen that the measured average absorptance across the broad bandwidth is ≈94% in comparison with ≈92% by the optical simulation. Obviously, the measured solar absorption value is a little higher than the design result, which is more favorable for photothermal applications. The slight spectral differences between measurement and simulation could be attributed to the thin film interface roughness (as shown in Figure 6B) and a certain degree of deviation in material optical parameters. On the other hand, in view of applications, SMAs should contain both the properties of high solar spectral absorptance and low thermal emissivity in the infrared region, according to the classical law of Planck's blackbody radiation. The spectral density of thermal radiation from a blackbody absorber can be defined by,^[^ ^47^ ^]^

(7) $$B_{\lambda }\;\left(\lambda ,T\right)=\frac{2hc^{2}}{\lambda^{5}}\;\frac{1}{e^{\frac{hc}{\lambda kT}-1}}\;$$

where *λ* is the wavelength, *T* is the absolute temperature of the blackbody absorber, *h* is Planck's constant, *c* is the speed of light in the vacuum, and *k* is the Boltzmann constant. According to the formula, we calculate the irradiance spectra at different temperatures in Figure S10 (Supporting Information). On the other hand, according to Kirchhoff's law at thermal equilibrium, the spectral emissivity and absorptance are equal at any specified temperature and wavelength.^[^ ^48^, ^49^ ^]^ We plot the simulated and measured emissivity spectra from 5 to 15 µm in Figure 6D. The great mass of the measured mid‐infrared emissivity rate is around 20%, which is quite beneficial for solar energy collection.^[^ ^50^ ^]^ Therefore, the SMA designed by the MST architecture demonstrates both high solar absorption and low thermal emissivity, which exhibits exceptional advantages over many state‐of‐the‐art SMAs, as illustrated in **Table** **1**.

**Table 1:** The performance comparison of state‐of‐the‐art solar metamaterial absorbers (SMAs)

Refs.	Absorptance^a)^	Average emissivity	Design scheme	Time
[10]	85% (measured)	**/**	Brute‐force simulation	Several days**/**weeks
[51]	87% (measured)	27% (measured)	Brute‐force simulation	
[52]	93% (simulated)	21% (simulated)	Iterative optimization	
[53]	94% (simulated)	**/**	Iterative optimization	
This work	94% (measured)	22% (measured)	MST deep learning	Several hours

^a)^ The average absorptance from visible to near‐infrared wavelengths.

### 2.5 Heating Performance of the SMA

**Figure 7:**
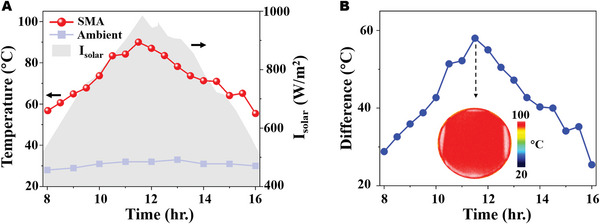
A) Timely solar radiation intensity and temperature curves of the solar metamaterial absorber (SMA) and ambient from 8:00 a.m. to 16:00 p.m. B) Temperature difference between the SMA and ambient within 8 h. The inset denotes the thermal images at 11:30 a.m.

We further perform a series of on‐site experiments to evaluate the application capability of our SMA (see more details in Figure S11, Supporting Information). **Figure** **7**A illuminates the timely solar radiation *I*_solar_, the temperature of SMA, and the ambient temperature from 8:00 a.m. to 16:00 p.m. under the sunlight (see more details in Figure S12, Supporting Information). One can see that *I*_solar_ increases from 8:00 a.m., reaches a one‐day peak, and decreases gradually. The temperature change trend of SMA is consistent with that of the daily solar radiation intensity while the ambient temperature remains at approximately 30 °C. Particularly, the temperature peak value of the SMA is about 90 °C and the maximum temperature difference between the ambient and SMA is up to 59 °C, as shown in Figure 7B. The thermal image at 11:30 a.m. shows the visualized contrast and indicates that the surface temperature of the SMA wafer is homogeneous.

**Figure 8:**
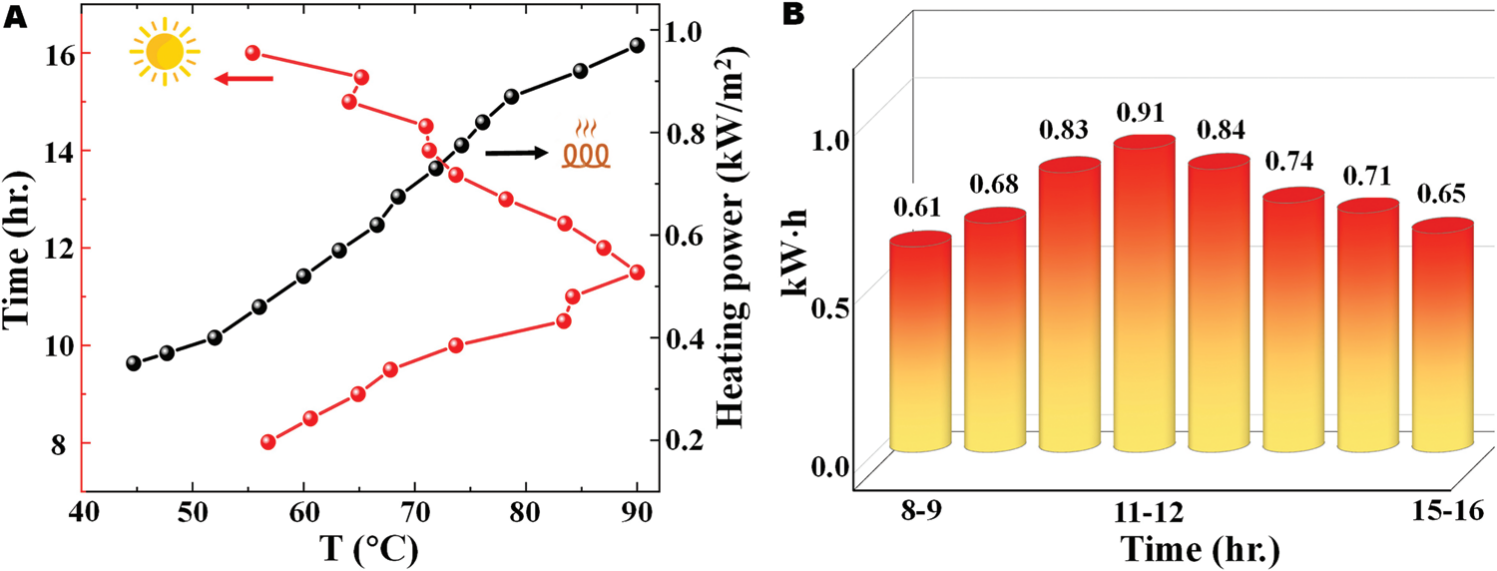
A) Temperature of the solar metamaterial absorber (SMA) as functions of local time and ohmic heating power. B) Collected energy (kW h) of 1 m^2^ SMA from 8:00 a.m. to 16:00 p.m.

With the aim to further evaluate the photothermal performance of SMA, we heat it with a polyimide heater to mimic the temperature‐rise effects of solar irradiation (see Figure S8, Supporting Information). As shown in **Figure** **8**A, the temperature of SMA is about 56 °C when the ohmic heating power is 460 W m^−2^, which corresponds to its temperature of about 56 °C under solar irradiation at 9:30 a.m. According to the pioneering work,^[^ ^49^, ^50^ ^]^ the measured power could be equivalent to the heating power obtained from sunlight. The equivalent heating power at other time points can be calculated by a similar way. Thus, the collected energy of our SMA from 8 a.m. to 4 p.m. is plotted in Figure 8B. The photothermal conversion from 11 a.m. to 12 p.m. demonstrates the highest performance and can collect about 0.91 kW h m^−2^ energy. The energy collected on this testing day is about 5.94 kW h m^−2^. The amount of energy collected in the corresponding month is estimated to be about 119.40 kW h m^−2^, assuming that the sunshine days for this month are 20 days based on the meteorological assessment in the city of the experiment.^[^ ^54^ ^]^ The estimated results in Figure S13 (Supporting Information) suggest that the SMA is capable of collecting about 1061 kW h m^−2^ energy annually, which is considerable and promising for solar power supply.

## 3 Conclusion

In summary, we establish an MST network based on the spectrum‐splitting scheme for the intelligent design of SMAs. Our method shows higher prediction precision by using much fewer training parameters than the classical MLP network for FNN. During the process of INN, it overcomes the overfitting problem of the CT network and provides the powerful physical insight for equivalent optical properties of SMAs. We further develop a flexible design tool that allows for quickly retrieving the structural parameters of SMAs from customer‐freely defined spectral requirements. The wafer‐scale SMA samples are fabricated based on the MST design. The samples exhibit higher solar light absorption and lower emissivity than many other state‐of‐the‐art SMAs. The outdoor experiments indicate the superior photothermal conversion performance of our SMA samples. Our method gives a flexible tool for intelligent design of light absorbers, and will also facilitate more applications in the field of metamaterials and metadevices.

## 4 Experimental Section

### Sample Fabrication

A 2‐inch and 0.5‐mm‐thick sapphire wafer (Zhongnuo New Materials Ltd. Co., China) was preprocessed by ultrasonic cleaning with organic solvents and deionized water (LvYin Reagent Ltd. Co., China). After drying, a series of target materials (Zhongnuo New Materials Ltd. Co.) were deposited on the wafer by an electron beam evaporator (DZS‐300, Shenyang Scientific Instrument Co., Ltd., China) according to the provided parameters. During the evaporation, the vacuum value was 4  × 10^−4^ Pa and the wafer temperature was less than 80 °C. The coating rate was about 0.3 nm s^−1^.

### Thermal Measurement

The outdoor solar radiation intensity *I*_solar_ was measured by a photoelectric solar radiation sensor (RS‐RA‐N01‐AL, Shandong Renke Control Technology Ltd. Co., China). The temperature of the sample was measured by a contact thermocouple thermometer (UT325, Uni‐Trend Technology Ltd. Co., China). The infrared images were recorded by an infrared camera (Testo‐869, Testo AG, Germany) with a spatial resolution of 160 × 120 at the spectrum ranging from 7.5 to 14 µm. The emissivity parameter was 0.22 in the measurement settings. A display‐power polyimide electric heater connected to a direct current supply (D69‐2058, Jiangsu Xiaochuangxin Electric Heating Appliance Co., China) provided heat generation.

### Optical Characterization

The absorptance spectrum was measured by the laboratory‐built system consisting of a light source, an integrated fiber‐optic probe, and a spectrophotometer (AvaLight, Avantes B.V., Netherlands). The fiber probe was perpendicular to the top of the samples above 4 mm during the experiment, as shown in Figure S14 (Supporting Information).

The infrared emissivity of the sample was measured by a Fourier infrared spectrometer (Nicolet iS50, Thermo Scientific, USA). The SEM (Sigma 300, Zeiss, Germany) was used to characterize the sample morphology.

### Numerical Simulations

The numerical simulations were calculated with the FDTD Solutions v8.13, Lumerical software. The plane waves along the *z*‐axis propagated to the solar absorber in the simulation. The boundary conditions along the *x*‐axis and *y*‐axis were periodic, and the boundary conditions along the z‐axis were perfectly matched layers. The optical parameters of Ge, Si, TiO_2_, Al_2_O_3_, SiO_2_, and MgF_2_ used in FDTD simulation were referred.^[^ ^55^ ^]^ In the simulation, the electromagnetic waves were introduced vertically incident on the SMA, and the normalized reflectance was collected with a frequency‐domain field and power monitor placed behind the excitation source.^[^ ^56^ ^]^

### Data Collection

A total of 141791 samples were collected using a desktop, among which 127590 were for the training samples, and 14201 were for the testing samples (Windows10 operation system, GeForce GTX 3080Ti GPU, Intel(R) Core(TM) i7‐10700K CPU @ 3.80 GHz 3.79 GHz and 16GB of RAM). The geometry ranges can be found in Table S4 (Supporting Information). The model was constructed based on the open‐source machine learning framework of PyTorch. The version used was Python3.8.

## Conflict of Interest

The authors declare no conflict of interest.

## Supporting information

Supporting Information

Supplemental Movie 1

Supplemental Movie 2

## Data Availability

The data that support the findings of this study are available from the corresponding author upon reasonable request.
