# Supplementary material for: Broadband Solar Metamaterial Absorbers Empowered by Transformer‐Based Deep Learning
Source: Adv Sci (Weinh). 2023 Feb 28;10(13):2206718. doi: 10.1002/advs.202206718 (PMC10161039; doi:10.1002/advs.202206718)
Supplement: Supplementary file 1 — Supporting Information [file ADVS-10-2206718-s003.pdf]

Broadband Metamaterial Solar Absorbers Empowered by Transformer-based Deep Learning

Wei Chen[#], Yuan Gao[#], Yuyang Li, Yiming Yan, Jun-Yu Ou, Wenzhuang Ma, Jinfeng Zhu [*]

W. Chen, Y. Gao, Y. Li, Y. Yan, Prof. J. Zhu

Institute of Electromagnetics and Acoustics and Key Laboratory of Electromagnetic Wave Science and Detection Technology, Xiamen University, Xiamen, Fujian 361005, China

# These authors contributed equally. *E-mail: nanoantenna@hotmail.com

Dr. J.-Y. Ou

Optoelectronics Research Centre and Centre for Photonic Metamaterials, University of Southampton, Highfield, Southampton, SO17 1BJ, UK

W. Ma

State Key Laboratory of Electronic Thin Films and Integrated Devices, National Engineering Research Center of Electromagnetic Radiation Control Materials, Key Laboratory of Multi-spectral Absorbing Materials and Structures of Ministry of Education, University of Electronic Science and Technology of China, Chengdu, Sichuan 610054, China

Details for Section 2.2 Model performance of MST

Fig. S1 shows the schematic of the multilayer perceptron (MLP) model for designing solar metamaterial absorbers (SMAs). The fully-connected layer sizes of forward neural network (FNN) are {6, 2500, 2500, 2500, 2500, 2500, 2500, 2500, 2500, 500}. The inverse neural network (INN) uses the multilayer perceptron with the layer sizes of {500, 1800, 1800, 1800, 1800, 1800, 1800, 1800,1024, 6}. The comparison of MLP and metamaterial spectrum transformer (MST) in the FNN is provided in Tab. S1.

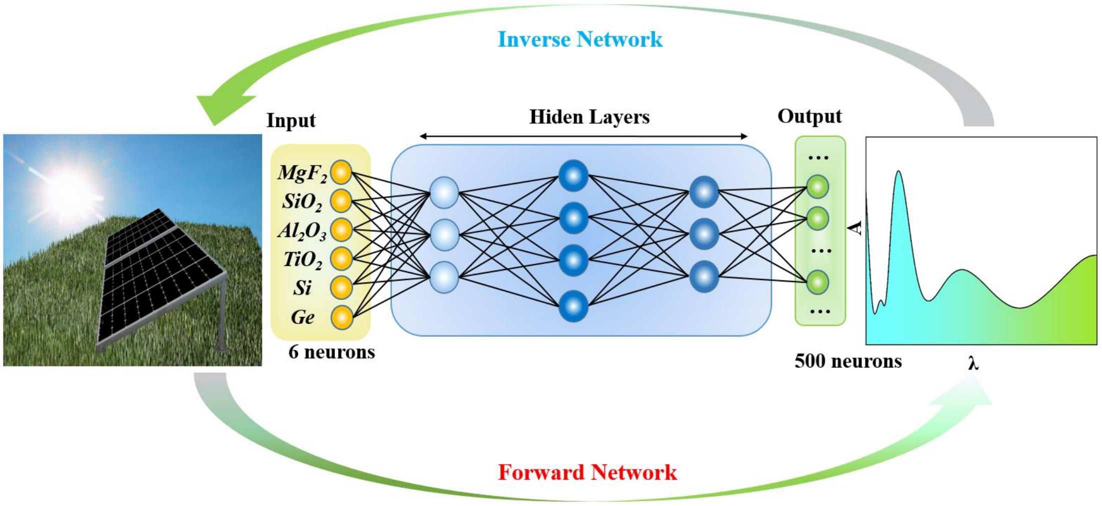

Fig. S1. The schematic of the MLP-enabled model for designing SMAs.

The two most pivotal hyperparameters in MST are the number of heads (*M*) and the number of layers (*L*). Parameter *M* stands for the number of head in the MST model, which can make the neural network learn different features of the physical model from different perspectives. Parameter *L* represents the number of layers, which provides a deeper learning capability. We conduct a lot of data tests to investigate the influence of parameters M and L on the MSE performance for the Learning curves of MST, as shown in Fig. S2(a-b). The MST model can only learn and extract a few features when *M* and *L* are 1, which causes high MSE, as shown in Fig. S2(a). With the increase of parameters *M* and *L*, the MSE is lower due to the MST has deeper learning capability to learn and extract features from different views. In fact, the use of *M*=8 and *L*=3 generates the minimum MSE in Fig. S2(a). During the training process, only partial heads significantly influence the training process and some heads may learn the iterative information. Simply extending this model with more layers could result in

gradient vanishing and causes bad prediction performance due to the interaction of residual connections and layer normalization. Moreover, the training parameters will become larger with *M* and *L* increase and lead to the problem of overfitting. Therefore, when we continue to increase the value of *M* and *L* after obtaining the minimum MSE, the MSE would not become lower but even increase. For instance, the MSE gets larger, when *M* = 9 and *L* =4. Therefore, we adopt the MST with *M*=8, *L*=3 and *M*=3, *L*=2 for FNN and INN, respectively. Furthermore, we randomly pick up two testing examples and study their performance in Fig. S2(c-d), showing that the structure prediction of the MST is better than that of MLP. The comparison of MLP and MST in the INN performance is provided in Tab. S2.

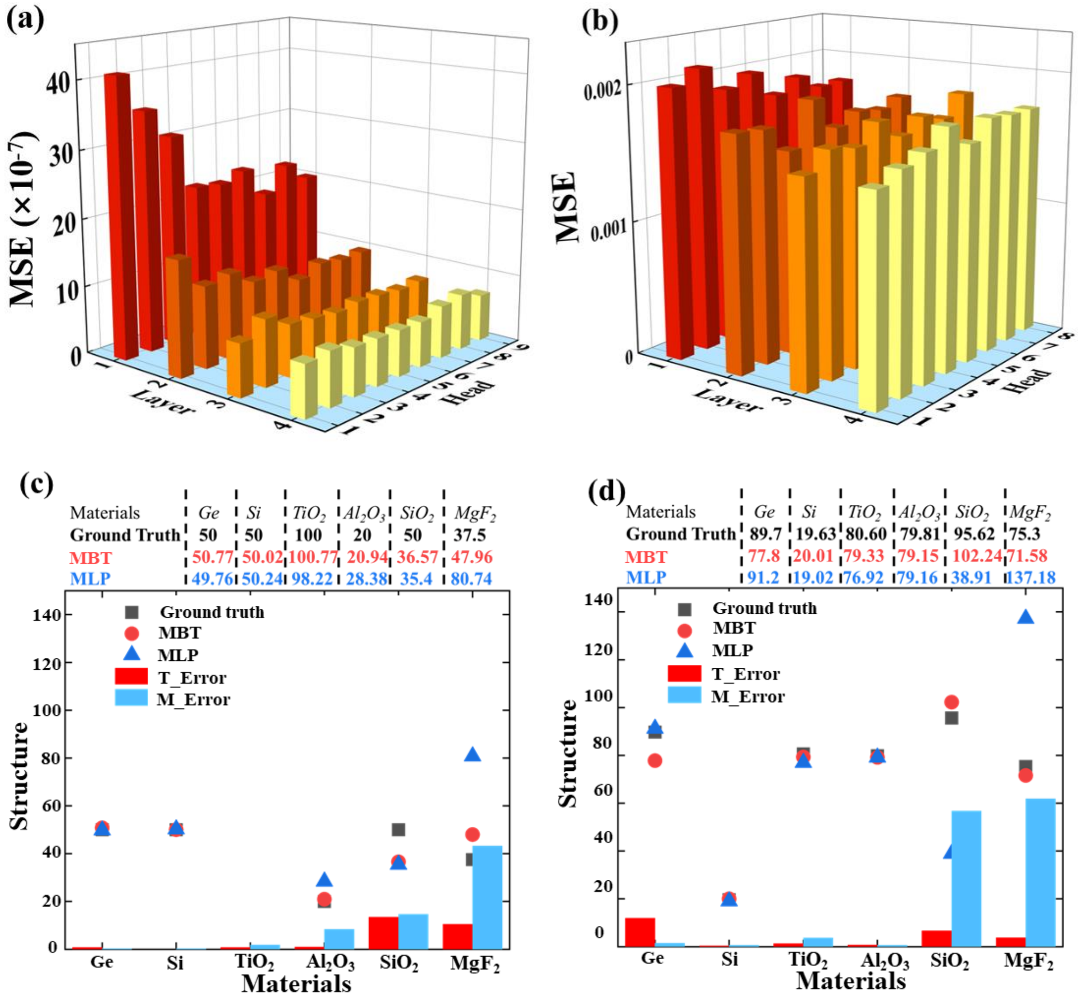

Fig. S2. (a) MSE of testing samples as a function of head number *M* and layer number *L* in FNN. (b) MSE of testing samples as a function of head number *M* and layer number *L* in INN. (c, d) Structure predicted by MLP and MST, their comparisons with the ground truth, and their absolute

errors.

Tab. S1 Forward performance demonstration of MLP and MST

Method	MSE	Training parameters
MLP	1.53×10^{-6}	51288000
MST (*M*=8, *L*=3)	6.63×10^{-7}	38149916
Critical reduction for improvement	56.7%	25.6%

Table S2 Inverse design performance of different deep learning models

**Method**	**Loss**	**Training parameters**
MLP	1.70×10^{-3}	25444774
MST (*M*=3, *L*=2)	1.52×10^{-3}	14001054
CT	2.0×10^{-3}	105227166

The equivalent phase velocity of electromagnetic waves in different loss-free and no-dispersion mediums can be expressed as $v=c/n$, where c is the light speed in the vacuum [1-3]. Fig. S3(a-b) exposits the nature of practically loss-free and non-dispersion for SiO_2 and MgF_2 [4]. Thus, the equal condition for electromagnetic wave propagation is,

$$T_{SiO_2}+T_{MgF_2}=T_{SiO_2}'+T_{MgF_2}'$$
$$(n_{SiO_2}\bullet d_{SiO_2})/c+(n_{MgF_2}\bullet d_{MgF_2})/c=(n_{SiO_2}\bullet d_{SiO_2}')/c+(n_{MgF_2}\bullet d_{MgF_2}')/c \quad (1)$$
$$n_{SiO_2}\bullet d_{SiO_2}+n_{MgF_2}\bullet d_{MgF_2}=n_{SiO_2}\bullet d_{SiO_2}'+n_{MgF_2}\bullet d_{MgF_2}'$$

Here, n_{SiO_2} and n_{MgF_2} are set to n=1.46 and 1.379. Therefore, this formula can be simplified as,

$$1.0587\bullet d_{SiO_2}+d_{MgF_2}=1.0587\bullet d_{SiO_2}'+d_{MgF_2}' \quad (2)$$

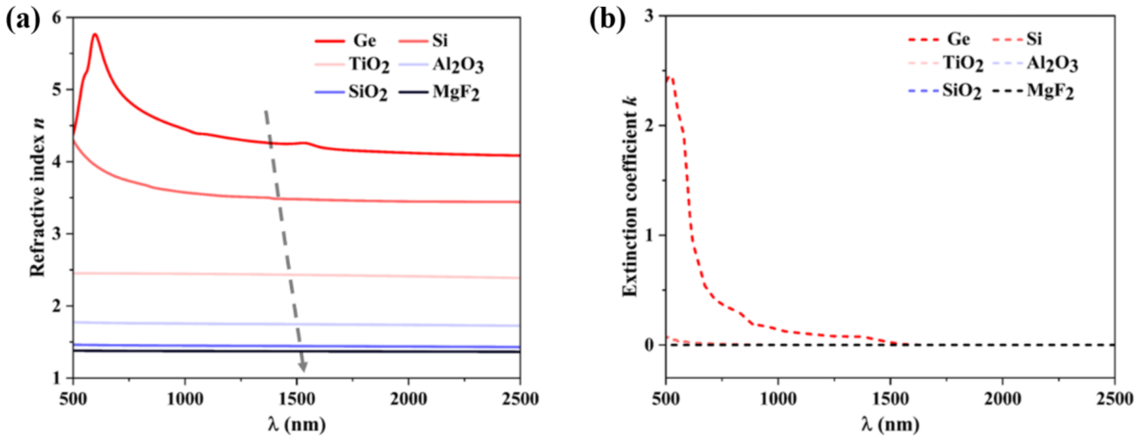

Fig. S3 Complex refractive index of the used natural materials. (A) Refractive index. (B)

Extinction coefficient.

Details for Section 2.3 Design of high-performance SMA and physical analysis

Our method can achieve the real-time on-demand design. We develop the customer-freely-defined multiple dots in the layout of the spectrum, which supports the cubic interpolation to provide the target spectrum as the input of inverse design. Since the prediction time is at the millisecond scale after training the entire network, the MST can output the real-time structural parameters according to customer-defined spectra. In Fig. S4(a), we freely manipulate the customer-defined green dots and quickly design a kind of SMA with an optical fiber sensing function [5]. In Fig. S4(b), we design a kind of SMA with two peaks within a specified wavelength range. The customer-defined inverse design can be performed to obtain a series of SMAs with different specific optical functions.

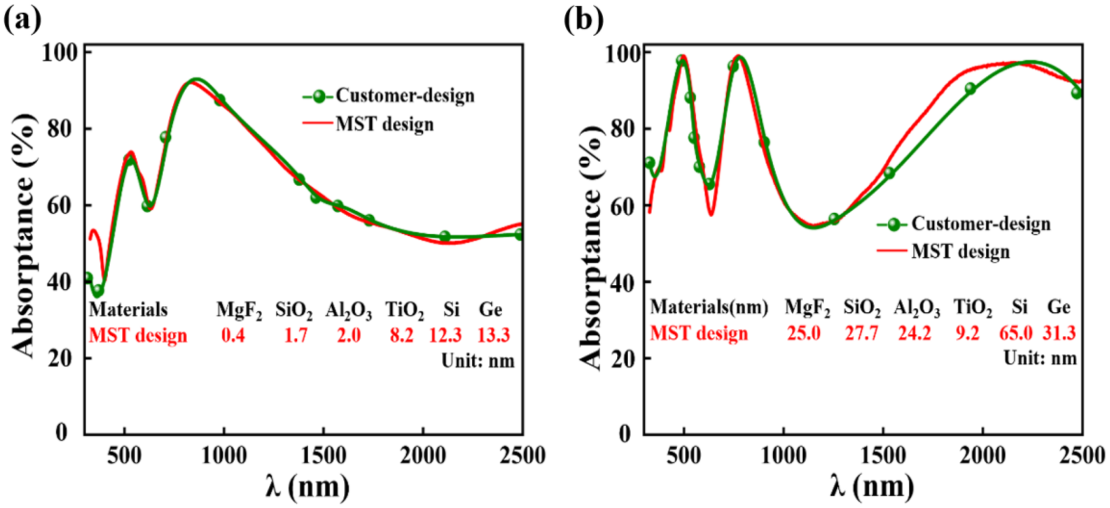

Fig S4 (A) On-demand design of metamaterial for optical fiber sensing. (B) On-demand design of optical metamaterial with two absorption peaks.

Furthermore, we use multiple customer-defined dots to confine the 100% absorptance spectrum as the input of inverse design. A real-time design result of SMA structural parameters and the corresponding absorptance spectrum can be generated, as shown in Fig. S5. The MST-predicting spectrum shows good agreement with the FDTD spectrum. The designed structure shows a high average absorptance of ~94% in a broad band from 500 to 2500 nm.

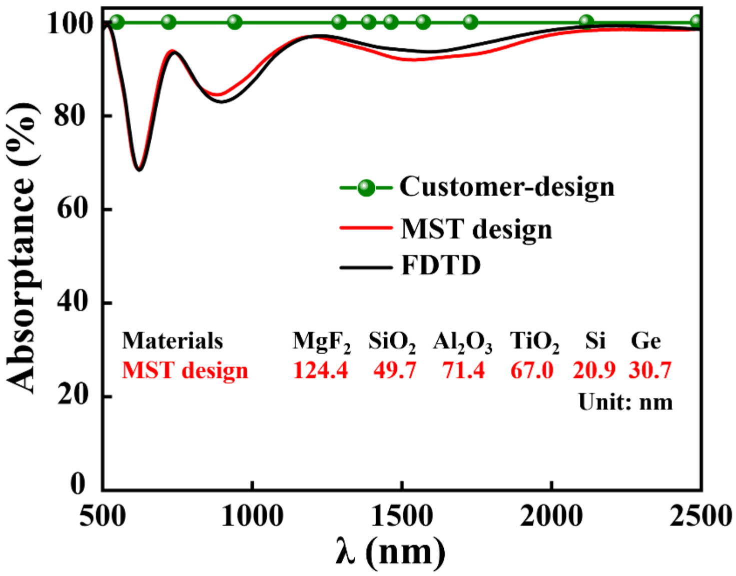

Fig. S5 SMA design for the on-demand spectrum of 100% absorption.

To demonstrate the advantages of the MST scheme, we adopt the particle swarm optimization (PSO) method as a proof-of-concept competitor. The PSO is a population-based stochastic optimization technique, inspired by the social behavior of flocks of birds or schools of fish, and has widely been widely used for various kinds of design optimization problems, including nanophotonic design [6]. The flowchart of the PSO method is shown in Fig. S6, where we set the maximal average absorptance of 500 to 2500 nm as the optimization goal. We employ 100 generations of 100 particles, which produced a total of 10000 particles. To highlight the relevance of the PSO algorithm, the genetic algorithm optimization process and reflection spectrum of all particles are created, as shown in Fig. S7. The optimization process lasts about 31 hours and the output absorptance is ~94%, which is very time-consuming and inefficient. However, when we give a spectrum with 100% absorptance, our MST method will give a solution closest to the 100% absorption spectrum at the millisecond scale, which also solves the optimization problem for absorptance. The performance comparison between the PSO method and the MBT approach is shown in Table S3, indicating the advantages of the MST scheme.

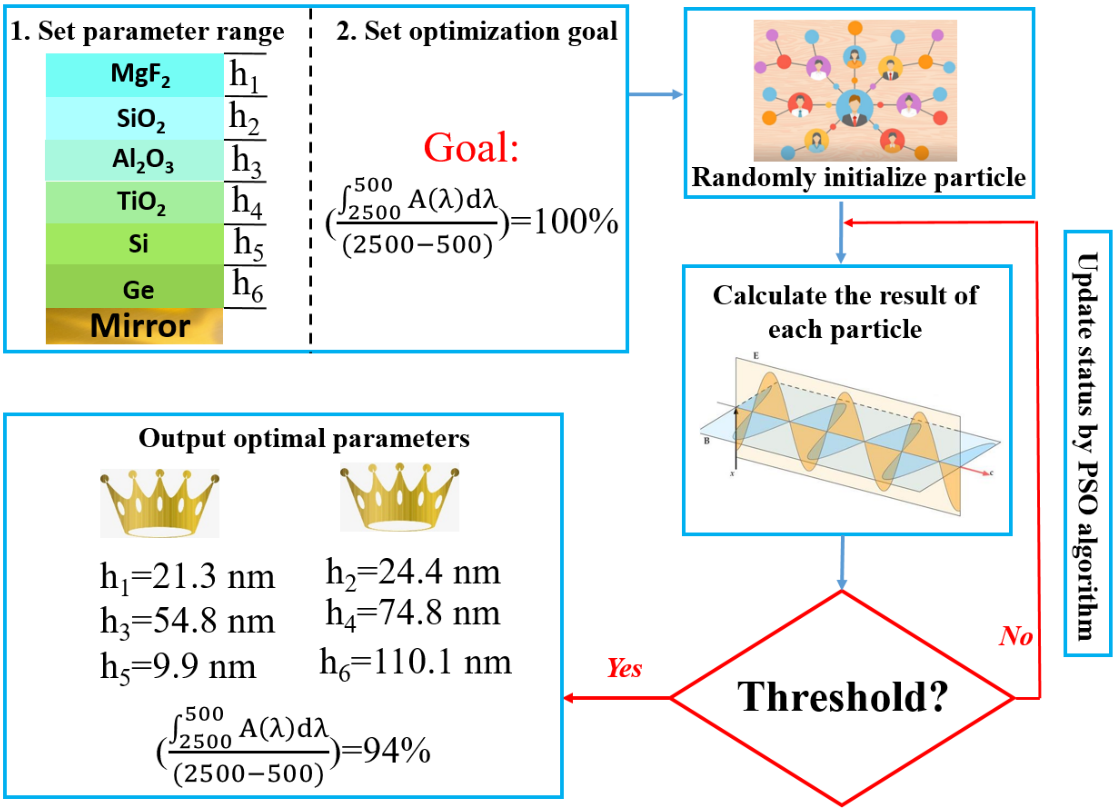

Fig. S6 Diagram illustrating the PSO design method of the SMA.

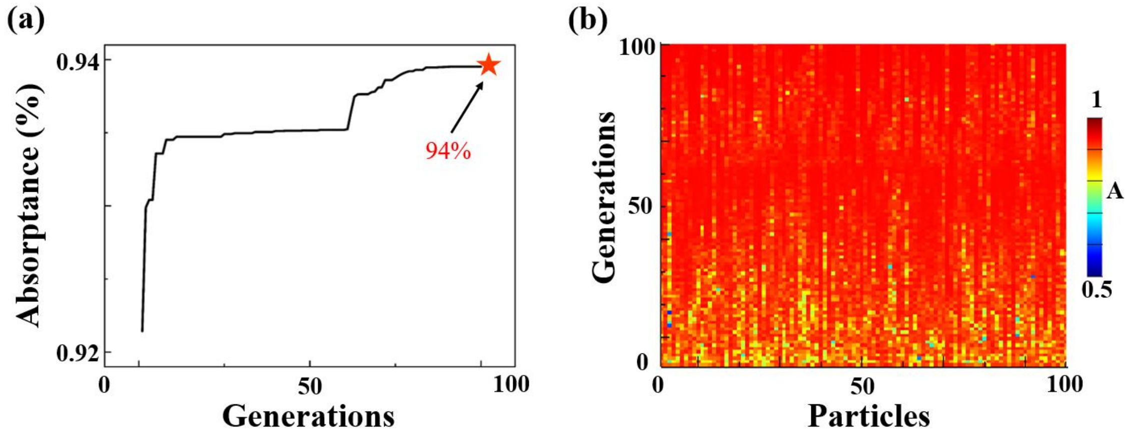

Fig. S7 (A) optimization process and (B) Absorptance spectrum of all particles by PSO method.

Table S3 The performance comparison between the PSO and MBT methods

Design scheme	Time	Average absorptance*
PSO method	~31 hours	~94%
MST approach	~1.6 hours	~94%

* The best average absorptance for the wavelengths from 500 to 2500 nm.

It’s worth noting that the majority of the solar energy is distributed in this short-wavelength range, namely, potential SMAs do not always pursue the maximum absorptance in such broadband. Therefore, we use multiple dots to adjust the absorptance spectrum until we get a high and steady solar absorption performance (See the Movie 2 in Supporting Information).

The optical admittances (inverse of the impedance) of this SMA sample at λ=811 nm and λ=1560 nm are plotted in Fig. S8.

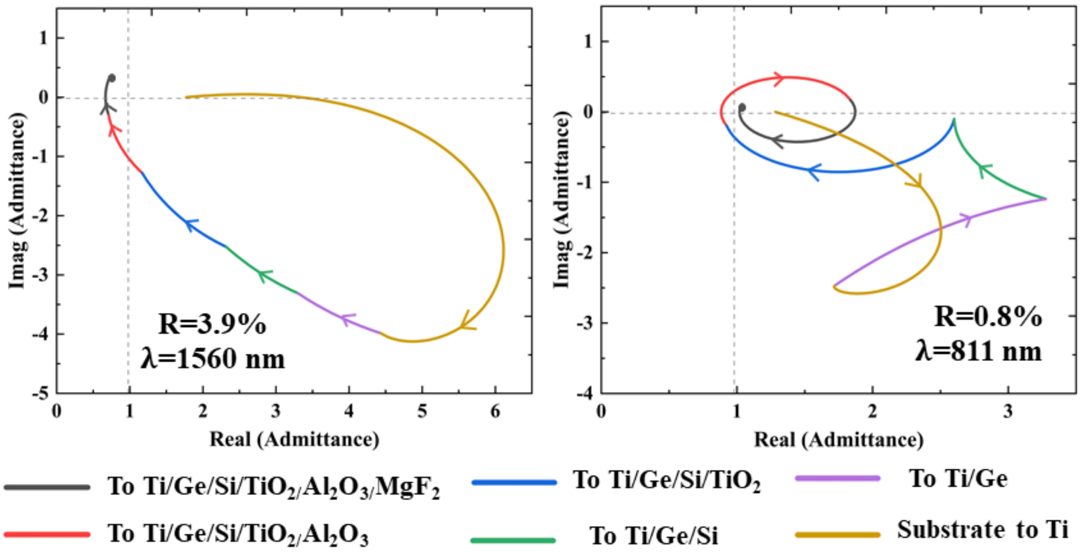

Fig. S8. The optical admittance locus of our SMA at λ = 811 nm and λ = 1560 nm.

It is well known that natural sunlight is an unpolarized source that could be oblique. We calculate the absorptance spectra with different incident angles under TM and TE modes using broadband fixed incident angle source technology in Fig. S9. The absorptance spectrum under an unpolarized source is from calculating absorptance spectra under TM and TE modes.

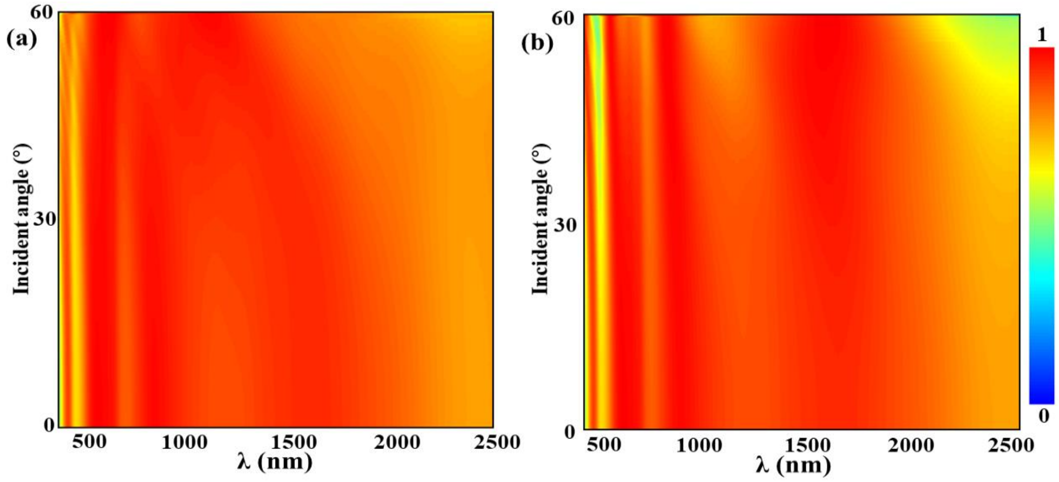

Fig. S9. Simulated absorptance spectrum under (a) TM and (b) TE mode swept with different incident angles.

Details for Section 2.4 Fabrication, characterization and optical measurement of the SMA

It can be seen from Fig. S10 that the maximum radiation occurs at about 6 and 10 μm at absolute temperatures *T* of 500 K and 300 K, respectively.

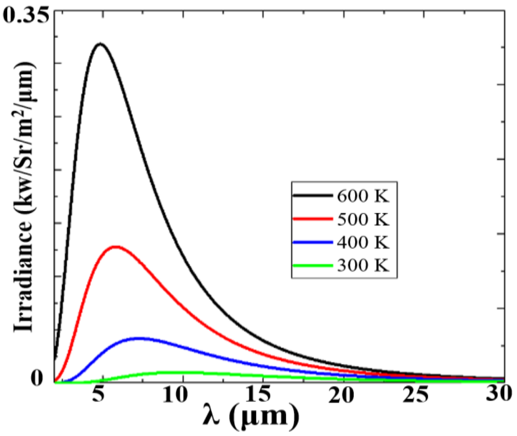

Fig. S10 Blackbody radiation spectra at T = 600 K, 500 K, 400 K, and 300 K.

Details for Section 2.5 Heating performance of the SMA

The experiment is took in Xiamen City, Fujian Province, China. The temperature (T), relative humidity, air quality (AQI), and wind direction of this day are plotted in Fig. S11(a) for reference. AQI is about 20, which means sunlight can ideally pass through the atmosphere and reach the ground. The distribution of solar energy in China is plotted in Fig. S11(b), where Amoy is 5000~5850 (middle level) and representative. Fig. S12(a) shows the outdoor photos of the absorber and handmade chamber for the photothermal measurement, consisting of a polystyrene and acrylic box. We use a contact coupling thermometer and an infrared thermal imager to measure and characterize the surface temperature of the fabricated SMA. To simulate the photothermal conversion of natural light, we test the device for indoor heating by the display-power polyimide heater where a thermocouple monitors the real-time temperature. The interference factors, such as a resistance tolerance of ±10%, are not considered.

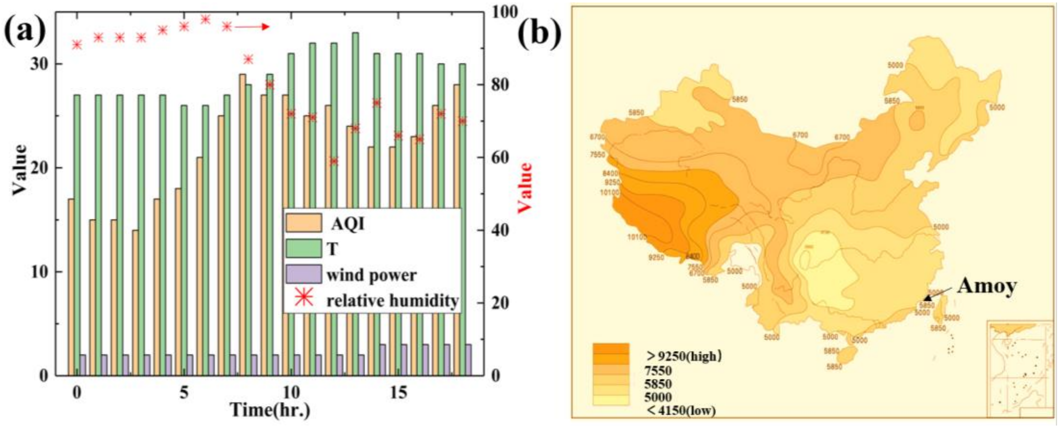

Fig. S11. (a) The temperature, relative humidity, air quality (AQI), and wind direction *vs.* time on 26 August 2021. Data source: China national meteorological center. (b) Distribution of solar energy in China(MJ·year/m^2). Data source: China meteorological administration

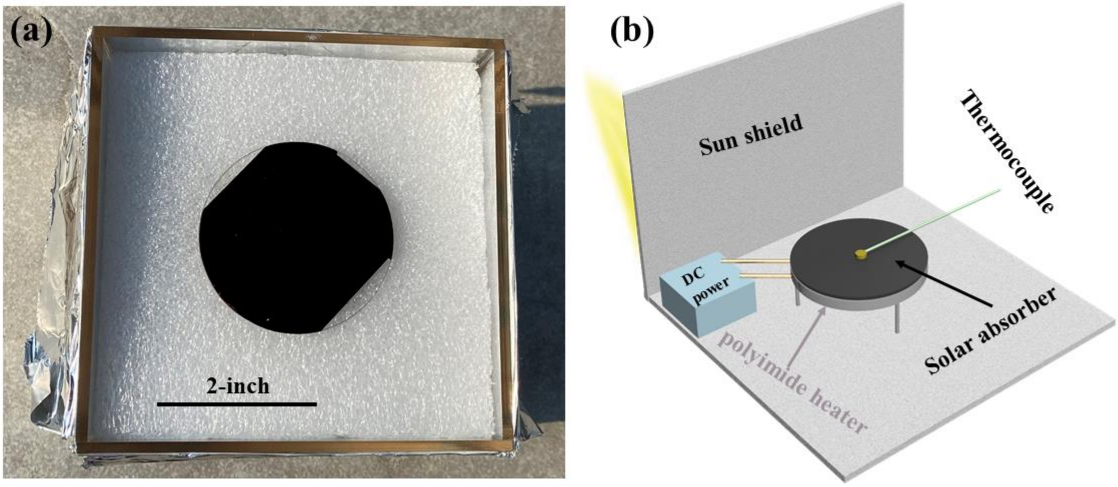

Fig. S12 (a) The digital images of the absorber and chamber. (b) The schematic diagram of the experiment to substitute solar radiation heating.

We refer to the monthly variation of solar radiation in Xiamen City [7] to objectively estimate the energy collected according to the assessment methods in Ref. [8] in Fig. S13(a), suggesting solar radiation in July is the highest and that in February is the lowest. We plot the estimated energy collected by the SMA every month in Fig. S13(b). In Fig. S13, August's data is derived from the measured results on 23 August while the rest are based on August's data and monthly variation of solar radiation.

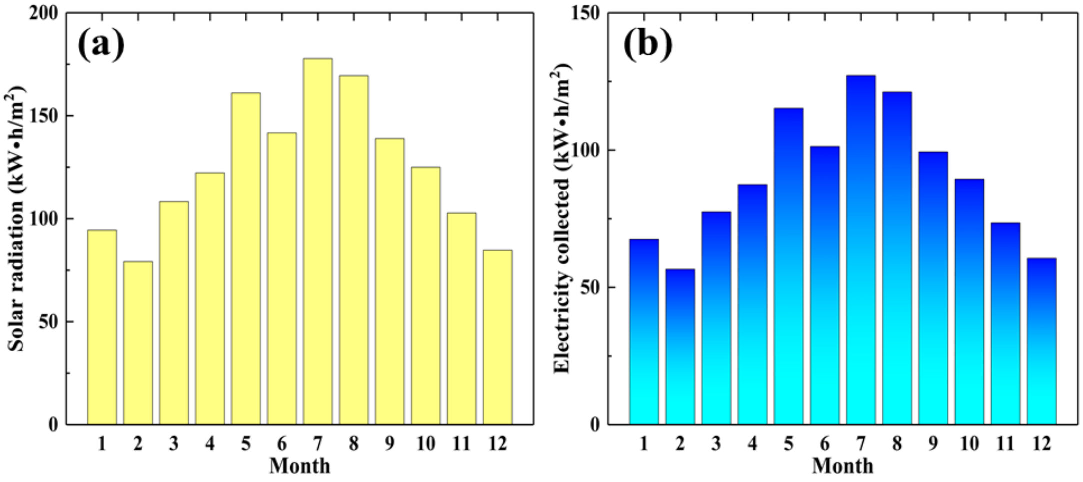

Fig. S13 (a) The monthly variation of solar radiation in Amoy [7]. (b) Electricity (energy) collected spectrum *vs.* months.

Details for Section 4 Material and methods

The schematics of the experimental apparatus for the absorptance measurement of the SMA are presented in Fig. S14.

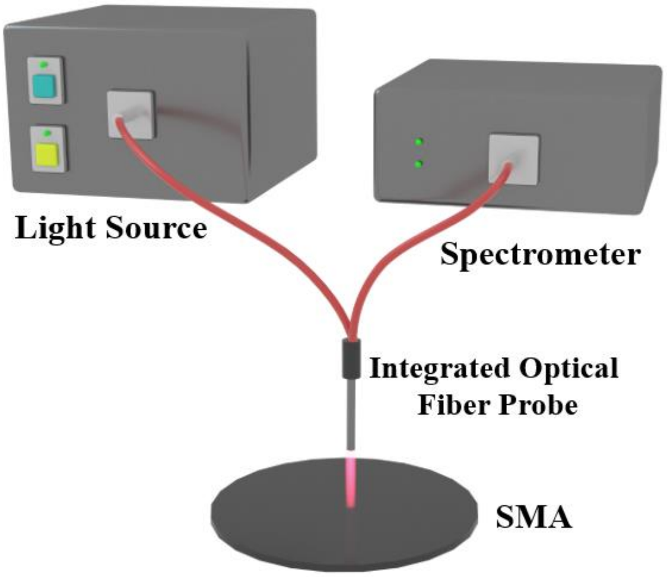

Fig. S14 Schematics of the experimental apparatus for the absorptance measurement of the SMA.

The geometry ranges of SMAs are shown in **Table S4**.

Table S4 Discussion ranges of geometric parameters

**Number**	**Materials**	**Range (nm)**
The 1st layer	Ge	0-100
The 2nd layer	Si	0-100
The 3rd layer	TiO_2	0-100
The 4rd layer	Al_2O_3	0-100
The 5rd layer	SiO_2	0-100
The 6rd layer	MgF_2	0-200

In order to demonstrate the generalization and validity of our method, the plasmonic stack metamaterials (PSMs) consisting of five alternating metal/dielectric layers are adopted as shown in Fig. S15(a), whose EM responses can not be calculated immediately using analytical equations. The optical constants of materials are from the literature [1]. With the aim to accelerate the generation of data sets, we develop the automatic parallel code based on rigorous coupled wave analysis. This powerful method can make the data collection process more efficient than the conventional electromagnetic simulation by commercial software. It is not necessary to redevelop the code of the deep learning (DL) model, and one only needs to update the neuron number of input and output. The training time of this DL model is about ~96 minutes. As illustrated in Fig. S15(b), the MST converges to a constant that is as low as 2.48×10^{-4}, which means our method can predict PSMs' electromagnetic responses with high accuracy. We randomly pick up a testing instance and draw the true absorptance spectrum and the absorptance spectrum predicted by MST in Fig. S15(c). The ground truth exhibits good agreement with the DL design. The PSMs example demonstrates our method can be widely applied to various metamaterials. More important, the inverse design function of deep learning also has potential applications in different fields that analytical equations are incapable.

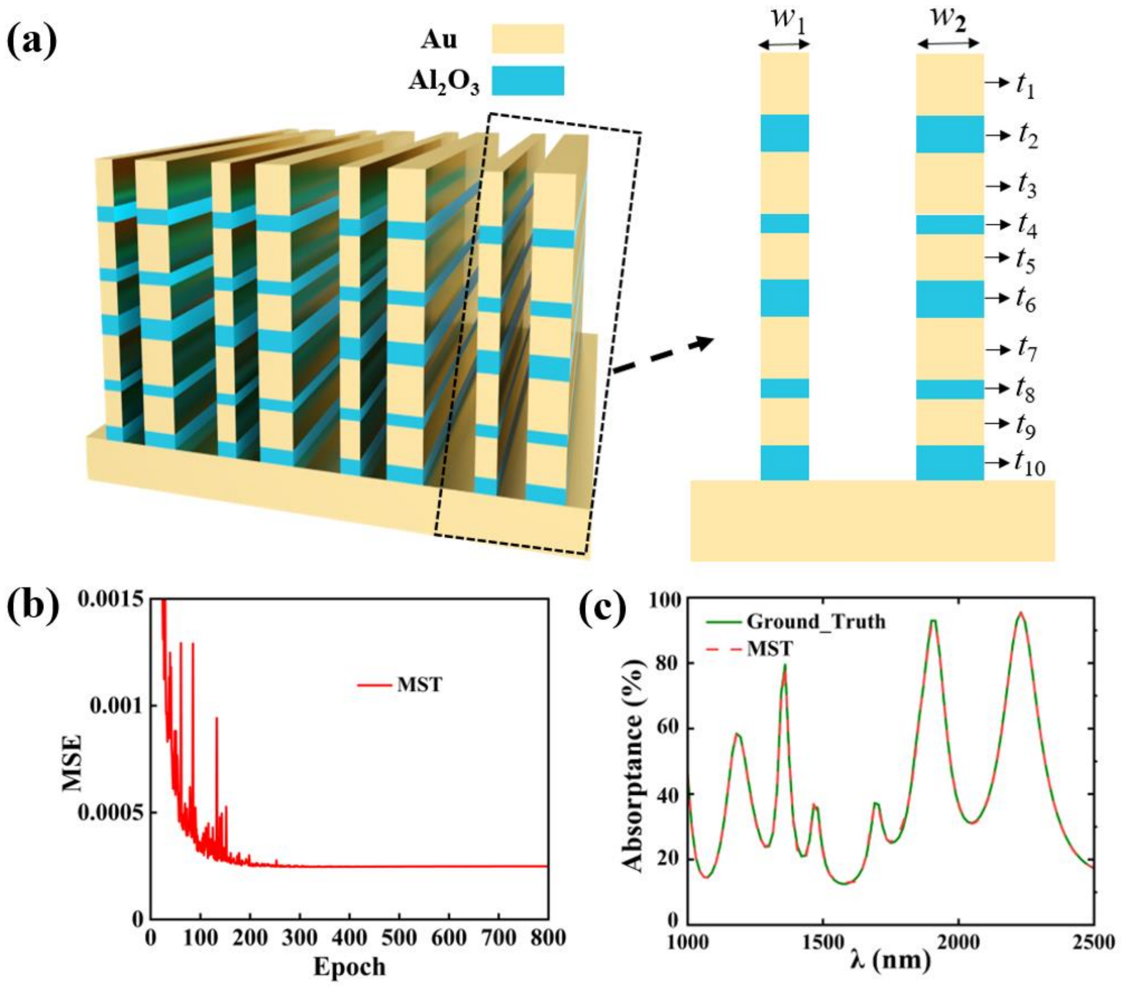

Fig. S15 (a) Schematic drawing of PSM with 12 nanostructure parameters in a metaunit. They are w_1, w_2, t_1, t_2, t_3, t_4, t_5, t_6, t_7, t_8, t_9, t_{10}. (b) The learning curves of forward design for the MST model. (c) Comparison between the MST-predicting spectrum and ground truth.

References

[1] Zhan, T. R.; Shi, X.; Dai, Y. Y.; Liu, X. H.; Zi, J. Transfer matrix method for optics in graphene layers. J. Phys.: Condens. Matter, 2013, 25, 215301.

[2] Szabo, Z.; Park, G.-H.; Hedge, R.; Li, E. P. Unique extraction of metamaterial parameters based on Kramers-Kronig relationship. IEEE Trans. Microw. Theory Tech., 2010, 58, 2646–2653.

[3] Smith, D.R.; Vier, D.C.; Koschny, T.; Soukoulis, C.M. Electromagnetic parameter retrieval from inhomogeneous metamaterials. Phys. Rev. E 2005, 71, 036617.

[4] Haynes, W.M.; Lide, D.R.; Bruno, T.J. CRC handbook of chemistry and physics. CRC press: Florida, FL, USA, 2016.

[5] Lan, G.; Wang, Y.; Ou, J.Y. Optimization of metamaterials and metamaterial-microcavity based on deep neural networks. Nanoscale Advances, 2022, 4, 5137-5143.

[6] Li, J.; Bao, L.; Jiang, S.; Guo, Q.; Xu, D.; Xiong, B.; Zhang, G.; Yi, F. Inverse design of multifunctional plasmonic metamaterial absorbers for infrared polarimetric imaging. Optics express, 2019, 27, 8375-8386.

[7] Wang, Y. M.; Wu, W. J.; Tang, Z. F. Evaluation of solar energy resources and analysis of solar energy application potential in Xiamen. The 32nd annual meeting of Chinese meteorological society, 2015.

[8] Raman, A. P., Anoma, M. A., Zhu, L., Rephaeli, E., Fan, S. Passive radiative cooling below ambient air temperature under direct sunlight. Nature, 2014, 515, 540-544.
