# Supplementary figures and images for: Broadband Solar Metamaterial Absorbers Empowered by Transformer‐Based Deep Learning
Source: Adv Sci (Weinh). 2023 Feb 28;10(13):2206718. doi: 10.1002/advs.202206718 (PMC10161039; doi:10.1002/advs.202206718)

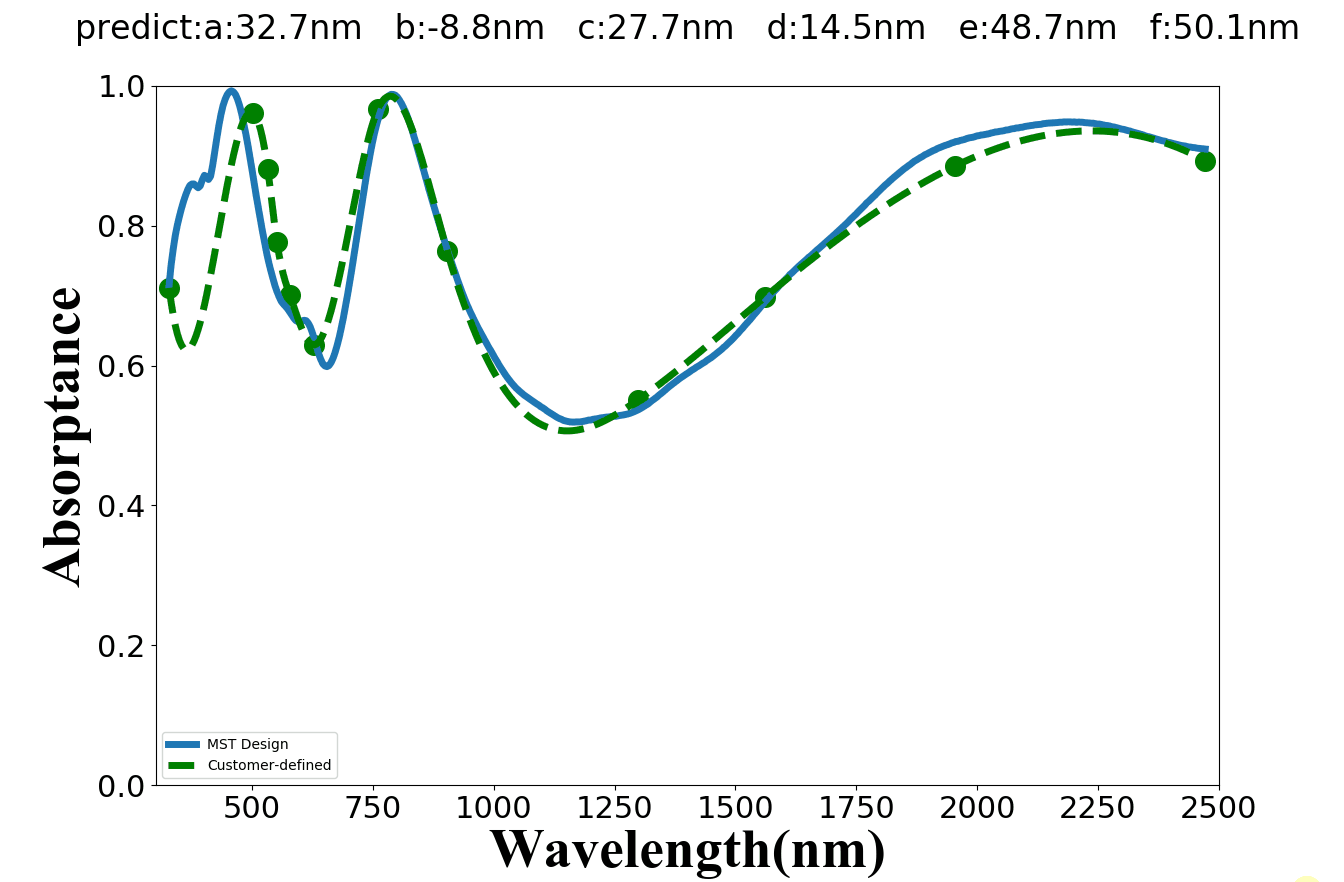

Supplement: Supplementary file 2 — Supplemental Movie 1 [file ADVS-10-2206718-s001.gif]

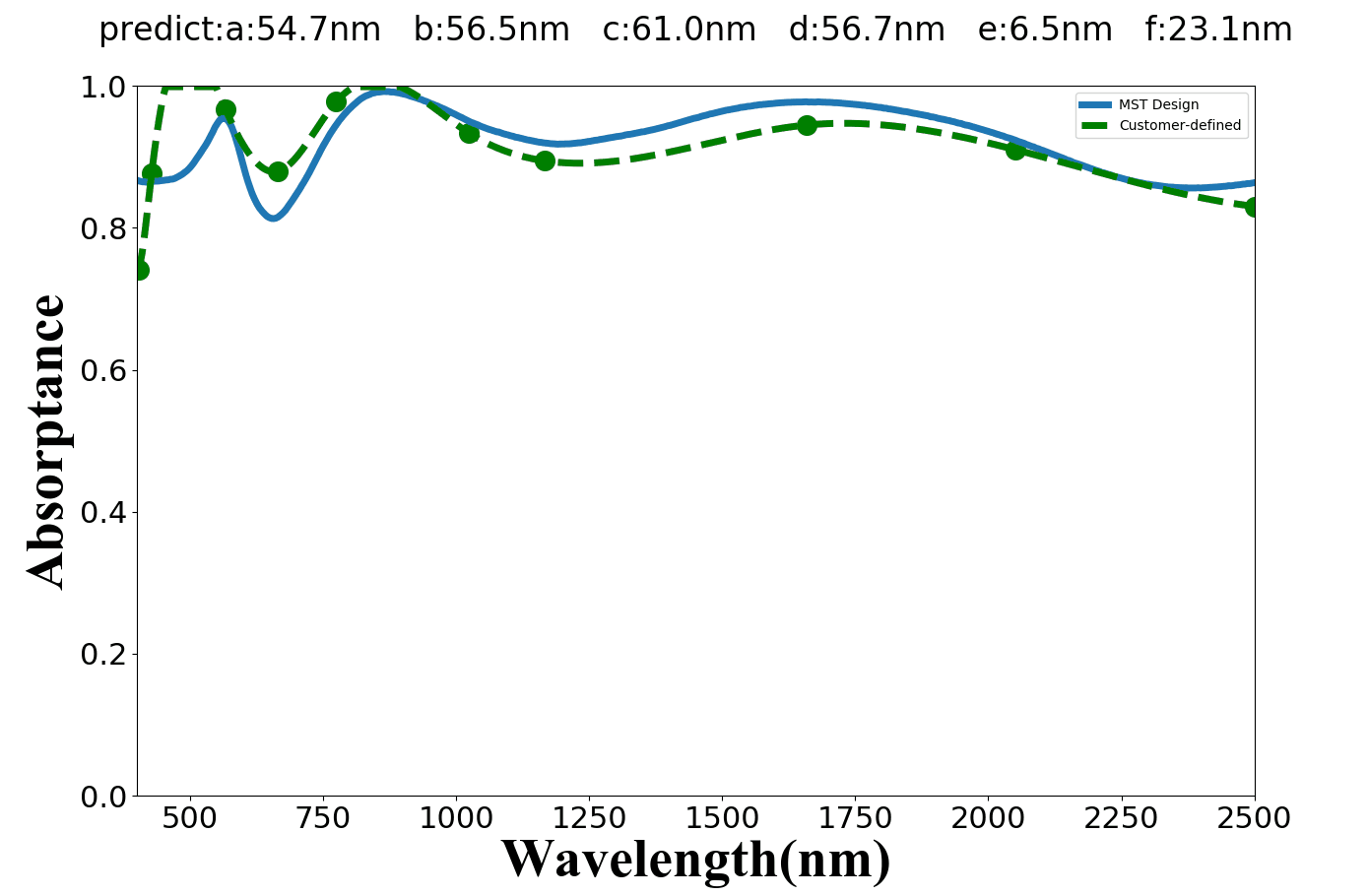

Supplement: Supplementary file 3 — Supplemental Movie 2 [file ADVS-10-2206718-s002.gif]
